# Preliminary findings of accelerated visual memory decline and baseline brain correlates in middle-age and older adults with autism: The case for hippocampal free-water

**DOI:** 10.3389/fnagi.2022.1029166

**Published:** 2022-11-11

**Authors:** Melissa J. M. Walsh, Edward Ofori, Broc A. Pagni, Kewei Chen, Georgia Sullivan, B. Blair Braden

**Affiliations:** ^1^College of Health Solutions, Arizona State University, Tempe, AZ, United States; ^2^Banner Alzheimer’s Institute, Phoenix, AZ, United States

**Keywords:** autism, aging, hippocampus, memory, autistic, MRI, DTI, fornix

## Abstract

Research aimed at understanding cognitive and brain aging in adults with autism spectrum disorder (ASD) is growing, but critical longitudinal work is scant. Adults with ASD struggle with tasks involving visual memory compared with neurotypical adults (NT). This may be related to differences in size or integrity of the hippocampus and its’ primary structural connectivity pathway, the fornix. The aim of this study was to describe preliminary findings of longitudinal aging trajectories in short- and long-term visual memory abilities in middle-age and older adults with ASD, compared with matched NT adults. We then evaluated baseline multi-modal imaging metrics of the hippocampal system, including the relatively novel metric of free-water, as potential correlates of longitudinal memory change in the ASD group. Middle-age and older adults with ASD (*n* = 25) and matched NT adults (*n* = 25) between the ages of 40 and 70 years were followed longitudinally at ~2-year intervals (range 2–5 years). Participants completed the Wechsler Memory Scale III Visual Reproduction task. Longitudinal mixed models were utilized to detect group differences in memory change with baseline age and sex as covariates. Hippocampal volume was measured *via* T1-weighted MRI images with FreeSurfer. Fornix fractional anisotropy and hippocampal and fornix free-water were measured from diffusion tensor imaging scans. Exploratory correlations were run between individual hippocampal system metrics and longitudinal slopes of visual memory change. There was a significant group by time interaction for long-term visual memory, such that middle-age and older adults with ASD declined faster than matched NT adults. There was no group by time interaction for short-term visual memory. Baseline hippocampal free-water was the only hippocampal system metric that correlated with long-term visual memory change in the ASD group. As one of the first longitudinal cognitive and brain aging studies in middle-age and older adults with ASD, our findings suggest vulnerabilities for accelerated long-term visual memory decline, compared to matched NT adults. Further, baseline hippocampal free-water may be a predictor of visual memory change in middle-age and older adults with ASD. These preliminary findings lay the groundwork for future prognostic applications of MRI for cognitive aging in middle-age and older adults with ASD.

## Introduction

Autism spectrum disorder (ASD) is a common neurodevelopmental condition that is associated with lifelong challenges which may worsen with age (Magiati et al., 2014; Howlin and Magiati, 2017). Further, recent evidence suggests middle-age adults with ASD are more likely to be diagnosed with early onset dementia compared to NT adults (Vivanti et al., 2021). These findings are supported by hypotheses that neurogenetic alterations associated with ASD may increase risk for age-related neurodegenerative conditions (Hickman et al., 2022). Further, we recently published preliminary longitudinal findings in middle-age and older adults with ASD suggesting accelerated hippocampal volume loss and short-term verbal memory decline (Pagni et al., 2022). Otherwise, characterizations of cognitive and brain aging trajectories for adults with ASD are primarily limited to cross-sectional studies, with inconsistent findings of safeguarded, parallel, or exacerbated brain and cognitive aging patterns (Geurts et al., 2016; Koolschijn et al., 2017; Braden and Riecken, 2019; Walsh et al., 2019; Bathelt et al., 2020; Tse et al., 2021). Longitudinal studies are the gold standard for aging research, especially in the context of ASD where cohort effects may present considerable confounds.

Declines in memory functioning are a hallmark of normal cognitive aging (Hoyer and Verhaeghen, 2006) and pathological aging associated with dementia (Salmon and Bondi, 2009). A recent meta-analysis spanning child to adult cohorts found that individuals with ASD have greater difficulty with visual long-term memory than verbal long-term memory. To date, only cross-sectional studies have examined patterns of age-related visual memory differences in ASD, revealing mixed evidence of age-related vulnerability to accelerated decline. For example, Geurts and Vissers (2012) found age patterns suggesting accelerated decline in short-term visual memory in middle-age and older adults with ASD compared to NT participants. However, in a larger follow up study using a broader young to older adult age range, adults with ASD showed age patterns suggesting safeguarding of short-term visual memory compared to NT adults (Lever and Geurts, 2016a). Yet, this safeguarding pattern was not replicated in a third follow-up study, which reported age patterns suggesting no difference in visual memory trajectories between ASD and NT groups (Torenvliet et al., 2022). Importantly, cohort effects and differing sample demographics may, in part, account for discrepancies across cross-sectional cognitive aging studies in ASD. The mixed findings to date highlight the importance of further research characterizing memory changes with age in ASD, in particular visual memory.

If adults with ASD are at greater risk for accelerated memory decline, as is suggested by the greater incidence of early onset dementia (Vivanti et al., 2021), developing prognostic biomarkers will be an important development for informing early diagnosis and precision treatment. The hippocampus is the primary structure underlying memory formation (Treves and Rolls, 1994; Zhang et al., 2010; Zheng et al., 2018) and neuropathological hippocampal markers have been extensively identified in ASD, ranging from decreased pyramidal cell size, diminished dendritic complexity and arborization, and altered GABAergic interneuron expression (Raymond, 1989; Bauman and Kemper, 2005; Lawrence et al., 2010). Such neuropathological processes may underlie memory challenges and altered hippocampal trajectories in aging adults with ASD. In NT adults, changes in hippocampal structure and function have been found to predict longitudinal declines in memory with age (Bettio et al., 2017). Thus, the hippocampus may be a candidate biomarker for predicting age-related memory decline in ASD. Previous research suggests adults with ASD have smaller hippocampi than NT adults (Van Rooij et al., 2018), and these findings extend to mid-to-older adult cohorts (Braden et al., 2017). Furthermore, a cross-sectional study of cortical thickness suggests that temporal regions associated with memory function may be vulnerable to accelerated atrophy in ASD (Braden and Riecken, 2019). Recent longitudinal evidence from our group showed accelerated hippocampal volume loss in middle age and older adults with ASD versus matched NT adults (Pagni et al., 2022). Thus, preliminary evidence indicates that hippocampal structural features (e.g., volume) may serve as promising candidate biomarkers for predicting cognitive aging risk in ASD.

The fornix is another brain structure that is critical for memory formation, and diffusion tensor imaging studies have demonstrated that microstructural integrity of the fornix (measured primarily *via* fractional anisotropy; FA) predicts age-related memory declines in healthy and pathological aging conditions like dementia (Douet and Chang, 2015). In cross-sectional studies of brain aging in ASD, evidence suggests that microstructural integrity of white matter tracts (Koolschijn et al., 2017) may be more sensitive to (cross-sectionally estimated) accelerated brain aging patterns in ASD compared to gray matter features (Geurts et al., 2016). For example, in investigations of cortical morphometry and white matter integrity in the same study sample, adults with ASD showed no differences in age patterns for morphometric features (e.g., cortical thickness, volume, surface area; Geurts et al., 2016), but white matter age patterns suggested accelerated declines in microstructural integrity in ASD (Koolschijn et al., 2017). Furthermore, we have previously shown reduced fornix FA in adults with ASD compared to NT participants (Pagni et al., 2022). Given the sensitivity of the fornix as a biomarker of cognitive aging risk in typical aging and dementia, as well as alterations to fornix integrity observed in adults with ASD, fornix microstructural features like FA may also serve as a promising biomarker for predicting memory aging trajectories in ASD.

Free-water is a newer DTI-derived metric which measures water molecules in extracellular space that do not have a directional dependence or other restrictions imposed by the cellular environment (Pasternak et al., 2009). Higher free-water values are thought to indicate atrophy and neuroinflammation (Pasternak et al., 2009). We were the first to establish that this novel, advanced DTI measure which is more sensitive for detecting neurodegeneration in mild cognitive impairment, Alzheimer’s disease, and Parkinson’s disease than conventional MRI metrics (Ofori et al., 2015a, 2017, 2019). These findings have since been replicated by other laboratories (Guttuso et al., 2018; Reas et al., 2018; Archer et al., 2021; Dzieciol et al., 2021; Shahid et al., 2022; Zhou et al., 2022). Most recently, Guttuso et al. (2021) demonstrated that baseline hippocampal free-water was a stronger predictor of follow-up cognitive status in Parkinson’s disease than hippocampal volume or conventional DTI metrics. To date, free-water has not been investigated in ASD, but may be sensitive for predicting age-related memory declines in ASD.

The primary aim of the present study was to characterize longitudinal aging trajectories in short-and long-term visual memory abilities in middle-age and older adults with ASD compared with matched NT adults. We hypothesized the ASD group would show accelerated declines in visual memory scores compared to NT adults. In an exploratory manner, we also evaluated baseline multi-modal imaging metrics of the hippocampal system, including the relatively novel metric free-water, as predictors for longitudinal memory change in middle-age and older adults with ASD. We hypothesized that baseline hippocampal system metrics would predict memory change in middle-age and older adults with ASD. Based on free-water’s sensitivity in mild cognitive impairment, Alzheimer’s, and Parkinson’s disease (Ofori et al., 2015a, 2017, 2019), we hypothesize this metric would show the strongest correlation with memory decline.

## Materials and methods

### Participants

**Figure 1:**
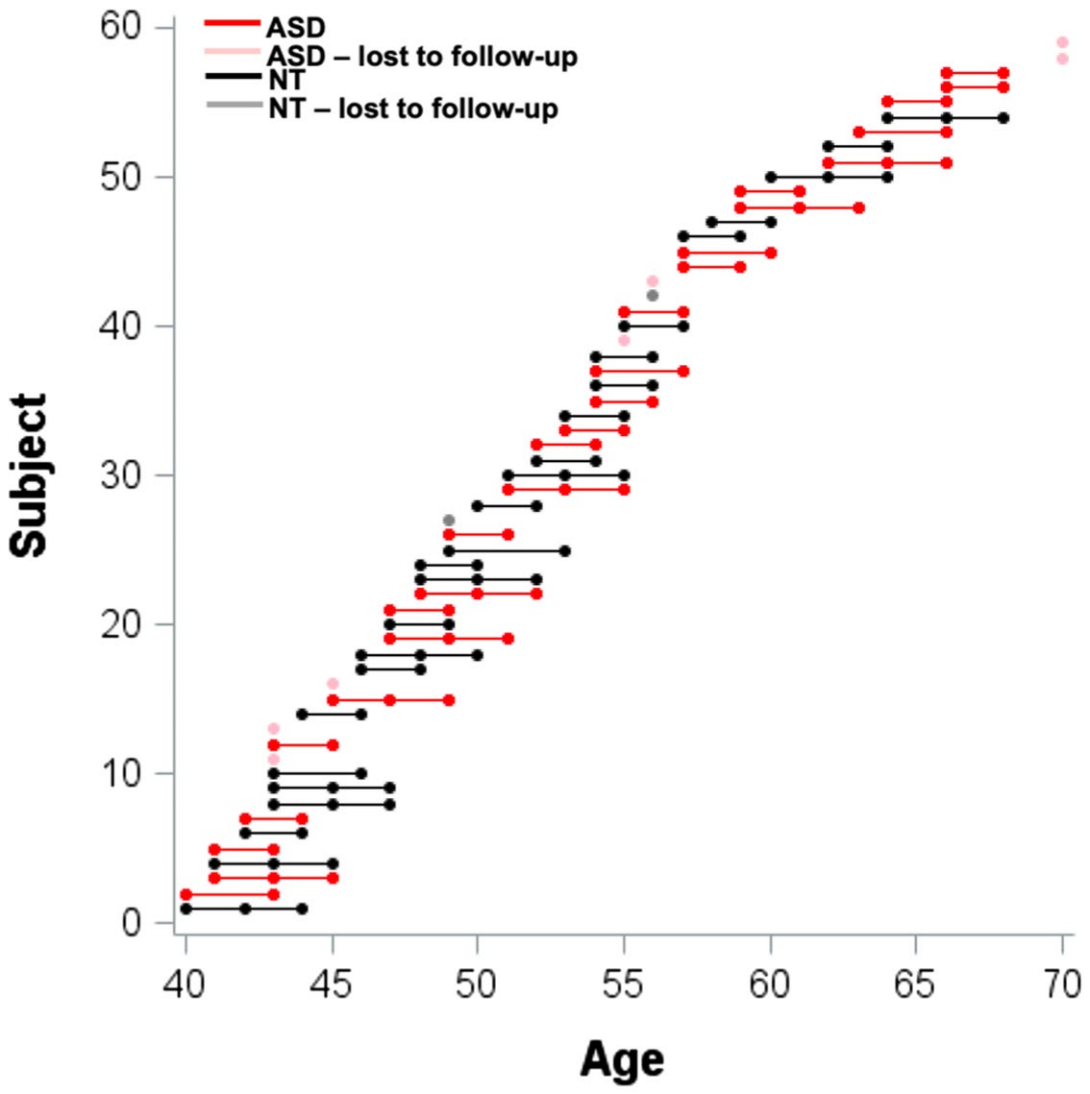
Distribution of longitudinal data as a function of age.

The study consisted of 25 middle-age and older adults (MA+) with ASD and 25 matched NT adults between the ages of 40 and 70 years who enrolled in a longitudinal study between the years 2014 and 2018. Participants were contacted *via* email or phone and completed follow-up visits at 2–3 year intervals. This represented an 86% retention rate for all MA+ participants who completed a baseline visit during these years (*n* = 58; Figure 1). Participants in both groups were recruited through flyers posted around Tempe, Arizona and Arizona State University and word of mouth. Some participants were additionally recruited from the Southwest Autism Research & Resource Center (SARRC) database. The SARRC database is voluntary and includes information about people who have been involved in previous clinical or research projects at SARRC. Participants in both groups underwent the same screening and enrollment procedures.

### Inclusion/exclusion criteria

Participants with ASD had their diagnosis formally verified at SARRC with the Autism Diagnostic Observation Schedule-2, module 4 (ADOS-2; Lord et al., 2012) and a brief psychiatric history interview administered by a research-reliable psychometrist. A score ≥ 7 on the ADOS-2 and an assessment by a psychologist with 25 years of ASD diagnostic experience confirmed DSM-5 criteria were met for their ASD diagnosis. NT participants were excluded if they had a first-degree relative with an ASD diagnosis, were suspected or confirmed to have an ASD diagnosis, or if they had a *T*-score > 66 on the Social Responsiveness Scale-2 Adult Self-Report (SRS-2; Constantino, 2012). Participants with ASD also completed the SRS-2 during screening (Table 1). Participants from both groups were excluded if their full-scale IQ score was < 70 on the Kaufman Brief Intelligence Test-2 (KBIT-2; Kaufman and Kaufman, 2004), they scored < 25 on the Mini Mental State Exam (MMSE; Folstein et al., 1975), or they self-reported neurological disease such as a stroke or dementia, head injury with loss of consciousness, known genetic disorders, or current use of seizure medications or illicit drugs. Psychiatric conditions were non-exclusionary because of high prevalence in ASD (Lever and Geurts, 2016b), and both psychiatric and general health conditions were tracked based on self-report of diagnoses and medications use (Supplementary Table 1). In total, 11 potential participants were excluded from the ASD group: seven did not meet criteria on the SRS-2, two did not meet the IQ criteria, and two reported head trauma with loss of consciousness. Eight potential participants were excluded for the NT group: four reported concerns for metal contraindications for the MRI, three reported head trauma with loss of consciousness, and one reported claustrophobia associated with MRI.

**Table 1:** Baseline participant demographics.

	NT (*n* = 25) Mean (± SD) Range	ASD (*n* = 25) Mean (± SD) Range	Statistics
Age (years)	50.00 (±6.78) 40–64	52.60 (±8.18) 40–66	*t*(48) = 1.22, *p* = 0.227
Sex (M/F)	21/4	21/4	*X*^2^(48) = 0.00, *p* = 1.000
Follow up duration (months)	34.76 (±11.32) 24–48	31.88 (±10.39) 24–48	*t*(48) = −0.94, *p* = 0.353
Number of follow up visits	2.40 (±0.50) 2–3	2.28 (±0.46) 2–3	*t*(48) = −0.89, *p* = 0.381
ADOS-2^a^ Social Affective	n/a	10.56 (±3.03) 7–19	n/a
Age at Diagnosis	n/a	46.96 (±13.68) 6–66	n/a
SRS-2^b^ Total t-score	46.00 (±5.72) 38–60	71.56 (±11.27) 51–88	*t*(35.6) = 10.109, *p* < 0.001^e^
MMSE^c^	29.40 (±0.91) 26–30	29.08 (±1.12) 26–30	*t*(48) = −1.110, *p* = 0.272
KBIT-2^d^ Composite	110.80 (±13.53) 89–141	111.00 (±13.66) 70–131	*t*(48) = 0.052, *p* = 0.959
DTI Motion (mm)	0.768 (±0.089) 0.598–0.936	0.771 (±0.100) 0.608–0.944	*t*(48) = 0.101, *p* = 0.920

a Autism Diagnostic Observation Schedule-2.

b Social Responsiveness Scale-2.

c Mini Mental State Exam.

d Kaufman Brief Intelligence Test-2.

e Levene’s test for equality of variances showed a significant group difference, thus statistics are presented after ‘equal variances not assumed’ adjustment.

### Visual memory

Participants performed the Wechsler Memory Scale III Visual Reproduction (WMS VR) task (Wechsler, 1997), typically the same day as the MRI, at Barrow Neurological Institute. The WMS VR is a task that assesses immediate (WMS VR I) and delayed visual recall (WMS VR II) whereby participants must recall details of a geometric line drawings and relative spatial relationships. The WMS VR II was administered 30 min after the WMS VR I. Raw scores for short-term immediate recall (WMS VR I) and long-term delayed recall (WMS VR II), estimating short-term memory and long-term memory respectively, were used for analyses.

### Magnetic resonance imaging

Baseline MRI data was acquired with a 3-Tesla Philips Ingenia MRI scanner with a maximum gradient strength of 45 mT/m at the Barrow Neurological Institute, St. Joseph’s Hospital and Medical Center, Phoenix, AZ. High-resolution, T1-weighted anatomical scans were captured using the following parameters: 3D magnetization prepared rapid acquisition gradient echo [MPRAGE] 256 × 256 in-plane resolution, 240 mm field of view (FOV); 170 sagittal slices 1.2 mm. T1-weighted MPRAGE images were used to generate hippocampal volumes in mm^3^ using the FreeSurfer automated parcellation.^1^ Left and right hippocampal volumes were divided by total intracranial volumes (TIV) mm^3^ to correct for individual differences in brain size. Hemisphere volumes were then averaged.

Gradient-echo echo-planar (EPI) DTI scans were collected over 4 min. Using the following parameters: echo time/repetition time (TE/TR) = 101/7,850 ms, bandwidth = 2,621 Hz/Px with a voxel size of 1.41 × 1.41 × 3 mm thick. DTI was obtained along 32 directions using a *b*-value of 2,500 s/mm^2^ in the axial direction with 3 mm slice resolution. DTI images were processed for eddy current, denoised, bias-field, and head motion corrected using FMRIB Software Library (FSL). Diffusion gradients are then compensated for rotations and non-brain tissue is removed. Using Advanced Normalization Tools (ANTs) non-linear transformations, the *b*0 was registered to the subject’s *T*1 in native space, then the transformation was applied to FA or free-water maps, and finally, volumes were nonlinearly registered to the MNI template. The quality of the dataset was evaluated with FSL quality control tools. This quality control involves using FSL for an automated non-parametric framework that corrects for distortions and signal loss. Motion was extracted from the affine motion and eddy current correction, and quantified by the average volume-to-volume head motion over each volume, which was relatively low given the short duration of the scan (4 min; Table 1).

Fractional anisotropy (FA) maps were calculated using custom written MatLab code. FA is a measurement of diffusion restriction that is thought to quantify axonal integrity and myelination of white matter (Pierpaoli and Basser, 1996; Ozarslan et al., 2005). High FA values indicate directional diffusion and low FA values indicate unrestricted diffusion. FA was chosen as our traditional DTI metric of interest because our previous paper demonstrated a higher effect size for FA differences in middle adulthood ASD vs. NT (Braden et al., 2017). The fornix ROI was from the John’s Hopkin’s University stereotaxic probabilistic white matter atlas that was generated from 81 normal subjects ranging from the ages of 18 to 59 (Mori et al., 2008). The body/bilateral cres values were averaged.

Free-water maps were calculated from a bi-tensor model with custom Matlab scripts (Ofori et al., 2015a, 2017, 2019). Briefly, the bi-tensor model predicts the signal attenuation in the presence of free-water contamination. It is the sum of attenuations contributed by two compartments: one that models free water, and a tissue compartment that models either gray matter or a single bundle of white matter. The FreeSurfer template was used for hippocampal ROI extraction, and the John’s Hopkin’s University atlas was used for the fornix ROI. One ASD participant was missing hippocampal free-water data due to technical error in template registration.

### Statistical analysis

Independent two-sample t-tests or chi-squared tests were conducted to examine baseline group differences in age, sex distribution, IQ (KBIT-2), global cognitive function (MMSE), DTI motion, and self-reported autistic traits (SRS-2). We used longitudinal mixed models to estimate diagnosis-differential trajectories of short-term and long-term visual memory change (WMS VR I and II) over time using SAS 9.4. Given literature suggesting between-subject variability in cognitive aging trajectories (Pagni et al., 2022), models included random intercepts and slopes. For these models, age was split into two separate variables per recommendations from Guillaume et al. (2014): between-subject (BS) age variability and within-subject (WS) age variability. Specifically, the mean age was calculated for each subject across visits and constituted the age-BS variable. Residual age from each subject’s mean age was then calculated at each time point, constituting the age-WS variable. The primary goal was to determine if there were differences in trajectories of change over time in ASD vs. NT. Thus, models were specified with the following predictors: diagnosis, age-WS, and age-WS*diagnosis with age-BS/sex as covariates of non-interest. For parsimony, an autoregressive covariance pattern model with constrained variances was used to model fixed effects. Given that little remains known about between subject variability in aging trajectories in ASD, we used an unstructured covariance pattern model for random effects. Proc sgplot was used to display marginal and subject-level predicted trajectories for visual short-term and long-term memory (WMS VR I and II). Confidence intervals were set at 95% for group-wise marginal trajectories.

In the presence of accelerated memory decline in ASD, exploratory correlations were run between baseline MRI metrics of the hippocampal system and longitudinal change slopes of visual memory metrics in the ASD group. As done previously with three time point longitudinal data (Persson et al., 2012), slopes were calculated from the first and final data point. The four baseline hippocampal system metrics were: (1) hippocampal volume, (2) hippocampal free-water, (3) fornix FA, and (4) fornix free-water, with hippocampal volume/free-water measuring grey matter structures and fornix FA/free-water measuring white matter structures. Correlations were exploratory; therefore, alpha was set at 0.05 with no correction for multiple comparisons. While adequate statistical power was difficult to determine given the novelty of longitudinal aging studies in ASD and associated MRI biomarkers, we estimated appropriate sample size from longitudinal aging studies in typical and other clinical populations. First, we have previously demonstrated baseline free-water of the substantia nigra correlates with longitudinal cognitive change in Parkinson’s patients with an effect size of 0.41 (Ofori et al., 2015b). Using a threshold of 0.80 power and 0.05 alpha (one tailed), a sample size of 22 participants was determined *via* G*Power 3.1.9.2. Second, in a longitudinal and brain aging study of typical elderly adults, hippocampal volume correlated with longitudinal change of a composite memory score with an effect size of 0.41 (Persson et al., 2012). Using the same thresholds, a sample size of 26 participants was determined.

## Results

### Demographics, DTI motion, and autistic traits

Groups were similar in age, sex distribution, IQ (KBIT-2), global cognitive function (MMSE), and DTI motion, but different on self-reported autistic traits (SRS-2; Table 1). Groups were also similar with respect to their follow up duration and total number of follow up visits (Table 1).

### Visual memory

**Figure 2:**
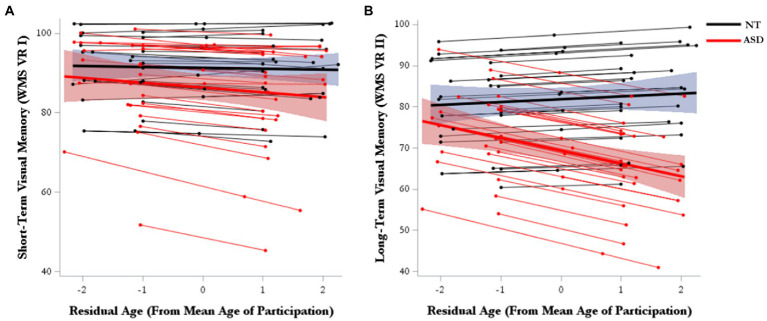
Model-predicted marginal and subject-level trajectories of change over time in **(A)** visual short-term memory and **(B)** visual long-term memory.

For short-term visual memory, there were no significant effects for either marginal trajectories of change (age-WS) or diagnosis-differential trajectories of change over time (age-WS*diagnosis; Table 2; Figure 2A). For long-term visual memory, there was a significant main effect of diagnosis, such that the ASD group had lower visual memory scores than the NT group (Supplementary Table 2). There was also a significant main effect of Age-BS, such that visual memory performance declined with increasing age. Furthermore, there was a significant effect of age-WS*diagnosis, such that ASD showed greater declines over time than NT groups (Figure 2B; Supplementary Table 2).

**Table 2:** Fixed effect estimates for longitudinal models of short-term and long-term visual memory.

		Estimate	Standard error	*df*	*t*-Value	*p*-Value
WMS VR I	Intercept	91.323	2.142	50	42.64	< 0.0001
	Diagnosis	−4.230	2.917	50.1	−1.45	0.153
	Age-WS	−0.344	0.555	21.3	−0.62	0.542
	Age-WS*Diagnosis	−1.030	0.827	21.9	−1.25	0.226
	Age-BS	−0.379	0.194	49.6	−1.96	0.056
	Sex	−3.900	3.919	51	−1.00	0.324
WMS VR II	Intercept	80.825	2.965	49	27.26	<0.0001
	Diagnosis	−9.646	4.055	50	−2.38	**0.021** ^*^
	Age-WS	0.655	1.163	43.4	0.56	0.576
	Age-WS*Diagnosis	−3.732	1.732	45.8	−2.16	**0.036** ^*^
	Age-BS	−0.838	0.275	49.6	−3.05	**0.004** ^*^
	Sex	−2.078	5.590	54.2	−0.37	0.712

* Bold indicates *p* < 0.05.

### Baseline hippocampal system correlates of memory decline in ASD

**Figure 3:**
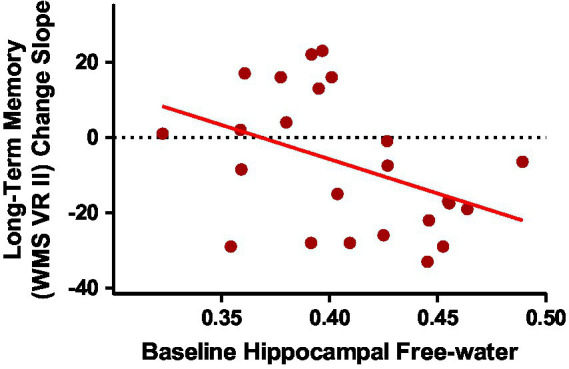
Baseline hippocampal free-water correlated with long-term visual memory change slopes in middle-age and older adults with ASD [*r*(24) = −0.416; *p* = 0.043].

Baseline group means and standard deviations for hippocampal system metrics can be found in Supplementary Table 3. As we have published previously on this cohort, the ASD group had smaller hippocampal volume and reduced fornix FA than the NT group (Pagni et al., 2022). Group differences in free-water did not reach statistical significance. Since the ASD group showed accelerated memory decline of long-term visual memory, exploratory correlations were run between baseline MRI metrics of the hippocampal system and longitudinal change slopes of visual memory metrics in the ASD group. Of the four hippocampal system metrics, only baseline hippocampal free-water significantly correlated with long-term visual memory change in the ASD group (Table 3; Figure 3).

**Table 3:** Baseline hippocampal system metrics correlations with visual long-term memory change slopes in middle-age and older adults with autism spectrum disorder (ASD).

	*df*	*r*	*p*-Value
Hippocampal volume %TIV	25	0.179	0.397
Fornix FA	25	0.316	0.125
Hippocampal FW	24	−0.416	**0.043** ^*^
Fornix FW	25	−0.268	0.198

* Bold indicates *p* < 0.05.

## Discussion

### Visual memory

In one of the first longitudinal investigations of memory change in MA+ ASD, our primary hypothesis was partially supported in that the ASD group showed accelerated declines in visual long-term memory, but not short-term memory, compared to matched NT adults. Visual memory has been the primary memory metric to show significant relationships with age in cross-sectional ASD studies, with conflicting findings of accelerated declining patterns in MA+ (Geurts and Vissers, 2012) to reduced declining patterns across a broad adult age-range (Lever and Geurts, 2016a). However, inconsistent with our findings, these papers highlight significant age relationships with short-term visual memory, not long-term. The most recent cross-sectional study by this group found parallel short-term visual memory trajectories in ASD vs. NT (Torenvliet et al., 2022), which is consistent with our longitudinal findings. In a meta-analysis spanning child to adult cohorts medium effect sizes were noted for long-term visual memory difficulties for individuals with ASD, in contrast to small effect sizes for verbal memory (Desaunay et al., 2020). Our preliminary longitudinal findings suggest long-term visual memory is a domain of vulnerability for accelerated decline in MA+ ASD.

### Baseline hippocampal system correlates of memory decline

Our exploratory hypothesis that baseline free-water would be the strongest correlate of memory decline in MA+ ASD was also supported. Free-water is a newer DTI-derived metric which is thought to indicate age-related and pathological processes of atrophy and neuroinflammation (Pasternak et al., 2009). Although the relationship between baseline hippocampal volume and fornix FA with long-term memory change were in the expected direction, baseline hippocampal free-water was the only metric that reached significance in MA+ ASD. Our findings agree with our previous work and others who have shown free-water is more sensitive for detecting neurodegeneration in mild cognitive impairment, Alzheimer’s, and Parkinson’s disease than traditional MRI metrics, such as hippocampal volume and FA (Ofori et al., 2015a, 2017, 2019; Guttuso et al., 2018; Reas et al., 2018; Archer et al., 2021; Dzieciol et al., 2021; Shahid et al., 2022; Zhou et al., 2022). We have previously shown baseline free-water in the substantia nigra predicts cognitive decline associated with Parkinson’s disease (Ofori et al., 2015b), and others have demonstrated that baseline hippocampal free-water was a stronger predictor of follow-up cognitive status in Parkinson’s disease than hippocampal volume or conventional DTI metrics (Guttuso et al., 2021). The present study is the first to extend free-water investigation to ASD. Our preliminary findings suggest free-water may be sensitive for predicting who is more likely to experience accelerated age-related memory decline associated with ASD. Future research should aim to determine if free-water is an *in vivo* proxy for and neuropathological hippocampal markers in ASD, such as decreased pyramidal cell size, diminished dendritic complexity and arborization, and altered GABAergic interneuron expression (Raymond, 1989; Bauman and Kemper, 2005; Lawrence et al., 2010).

Interestingly, the group differences in baseline hippocampal and fornix free-water did not reach significance. In contrast, MA+ ASD had significantly smaller hippocampi and reduced fornix FA at baseline, compared to NT participants, but these metrics did not correlate with subsequent memory change in MA+ ASD. Taken together, these preliminary findings suggest there is more variability in hippocampal/fornix free-water, than volume/FA, but free-water may be more biologically meaningful for memory abilities in MA+ ASD. There is consistent literature on reduced hippocampal size associated with ASD across the lifespan, which has been replicated in a large sample (Van Rooij et al., 2018). More research on hippocampal free-water across the broad age ranges in ASD is needed to shed light on the developmental trajectory and relationship with memory abilities across the life course.

### Limitations

First, this is a small sample and findings should be interpreted as preliminary with need for replication in larger samples. Second, sex is an important factor in cognitive and brain function in ASD (Bölte et al., 2011; Walsh et al., 2021). We included sex as a covariate in longitudinal models, however, there were not enough women in the sample to investigate the role of sex differences in MA+ memory decline. We began our longitudinal study in men, but now have ~ 2:1 representation of male:female in our study to determine the role of sex in cognitive and brain aging in ASD in future investigations. Third, uneven occurrence of mental health conditions across groups (Supplementary Table 1) could interact with cognitive decline, which warrants future investigation in larger studies. Lastly, about one third of those diagnosed with ASD have comorbid intellectual disability (ID). Our study includes only cognitively-able adults. Future research is warranted to determine cognitive and brain aging trajectories in adults with ASD and comorbid ID.

## Conclusion

In one of the first longitudinal cognitive aging investigations in MA+ ASD, findings suggest vulnerabilities for accelerated long-term visual memory decline, compared to matched NT adults. Further, baseline hippocampal free-water may be a predictor of memory decline in middle-age and older adults with ASD. These preliminary findings require replication in larger samples. Future prognostic applications of MRI for cognitive aging with ASD are especially needed given the increased rates of dementia in this population.

## Data availability statement

The datasets generated for this study will be made available upon request.

## Ethics statement

The studies involving human participants were reviewed and approved by Arizona State University Institutional Review Board. The patients/participants provided their written informed consent to participate in this study.

## Author contributions

BB oversaw all aspects of the study including design, recruitment, data collection and analyses, and manuscript preparation. MW led recruitment, data collection and analyses, and manuscript preparation. EO led DTI analyses and assisted with manuscript preparation. BP assisted with recruitment, data collection and analyses, and manuscript preparation. KC and GS assisted with data analysis and manuscript preparation. All authors contributed to the article and approved the submitted version.

## Funding

This study was funded by the Arizona Biomedical Research Commission (grant/award number: ADHS16-162413); U.S. Department of Defense (grant/award number: AR140105); National Institute of Mental Health (grant/award numbers: F31MH122107 and K01MH116098); National Center for Complementary and Integrative Health (grant/award number: F31AT010976).

## Conflict of interest

The authors declare that the research was conducted in the absence of any commercial or financial relationships that could be construed as a potential conflict of interest.

## Publisher’s note

All claims expressed in this article are solely those of the authors and do not necessarily represent those of their affiliated organizations, or those of the publisher, the editors and the reviewers. Any product that may be evaluated in this article, or claim that may be made by its manufacturer, is not guaranteed or endorsed by the publisher.

## Footnotes

^1^ https://surfer.nmr.mgh.harvard.edu
