# Supplementary material for: Preliminary findings of accelerated visual memory decline and baseline brain correlates in middle-age and older adults with autism: The case for hippocampal free-water
Source: Front Aging Neurosci. 2022 Nov 11;14:1029166. doi: 10.3389/fnagi.2022.1029166 (PMC9694823; doi:10.3389/fnagi.2022.1029166)
Supplement: Supplementary file 1 [file Table_1.DOCX]

Supplementary Material

**Supp. Table 1. Psychiatric and Health Conditions**

|  | **ASD (n; % sample)** | **NT (n; % sample)** |
| --- | --- | --- |
| **Any Psychiatric/Psychological Problem** | **8 (30.8%)** | **1 (4.2%)** |
| Depression | 6 (23.1%) | 1 (4.2%) |
| Anxiety | 2 (7.7%) | 0 (0%) |
| ADHD/ADD | 2 (7.7%) | 1 (4.2%) |
| Learning Disorder/Dyslexia | 1 (3.8%) | 0 (0%) |
| Bipolar/Mood Stabilizing | 1 (3.8%) | 0 (0%) |
| **Any General Health Problem** | **7 (26.9%)** | **5 (20.8%)** |
| Cardiovascular | 3 (11.5%) | 2 (8.3%) |
| Joint/Bone | 0 (0%) | 0 (0%) |
| Endocrine | 1 (3.8%) | 2 (8.3%) |
| Gastrointestinal | 2 (7.7%) | 0 (0%) |
| Sleep | 0 (0%) | 0 (0%) |
| Allergies/Asthma | 1 (3.8%) | 2 (8.3%) |
| Back | 0 (0%) | 0 (0%) |
| Migraines | 2 (7.7%) | 0 (0%) |
| Cancer remission/Tumor | 0 (0%) | 0 (0%) |

| **Supp. Table 2. Visual reproduction test means and standard deviations** | | | | |
| --- | --- | --- | --- | --- |
|  | **WMS VR I** | | **WMS VR II** | |
|  | **ASD** | **NT** | **ASD** | **NT** |
| **Time 1 (n=25)** | 88.72 (13.51) | 93.84 (7.55) | 74.00 (18.19) | 80.92 (17.05) |
| **Time 2 (n=25)** | 83.44 (13.99) | 87.84 (11.53) | 68.40 (19.36) | 79.48 (16.97) |
| **Time 3 (n=16)** | 88.71 (18.51) | 96.56 (8.37) | 56.14 (28.16) | 91.33 (12.96) |

| **Supp. Table 3.** **Baseline group means and standard deviations for hippocampal system metrics** | | | |
| --- | --- | --- | --- |
|  | **ASD (n=25)** | **NT (n=25)** | **t-test** |
| **Hippocampal volume %TIV** | 0.335 (±0.037) | 0.366 (±0.039) | t(48)=2.906; **p=0.006*** |
| **Fornix FA** | 0.424 (±0.038) | 0.443 (±0.025) | t(48)=2.112; **p=0.040*** |
| **Hippocampal FW^a^** | 0.408 (±0.042) | 0.395 (±0.037) | t(47)=1.159; p=0.252 |
| **Fornix FW** | 0.617 (±0.098) | 0.571 (±0.087) | t(48)=1.757; p=0.085 |
| **^a^** One ASD participant was missing hippocampal FW data; *p<0.05 | | | |
